# Supplementary material for: Production of santalenes and bergamotene in Nicotiana tabacum plants
Source: PLoS One. 2019 Jan 4;14(1):e0203249. doi: 10.1371/journal.pone.0203249 (PMC6319812; doi:10.1371/journal.pone.0203249)
Supplement: S1 Table — (PPTX) [file pone.0203249.s001.pptx]

## Slide 1
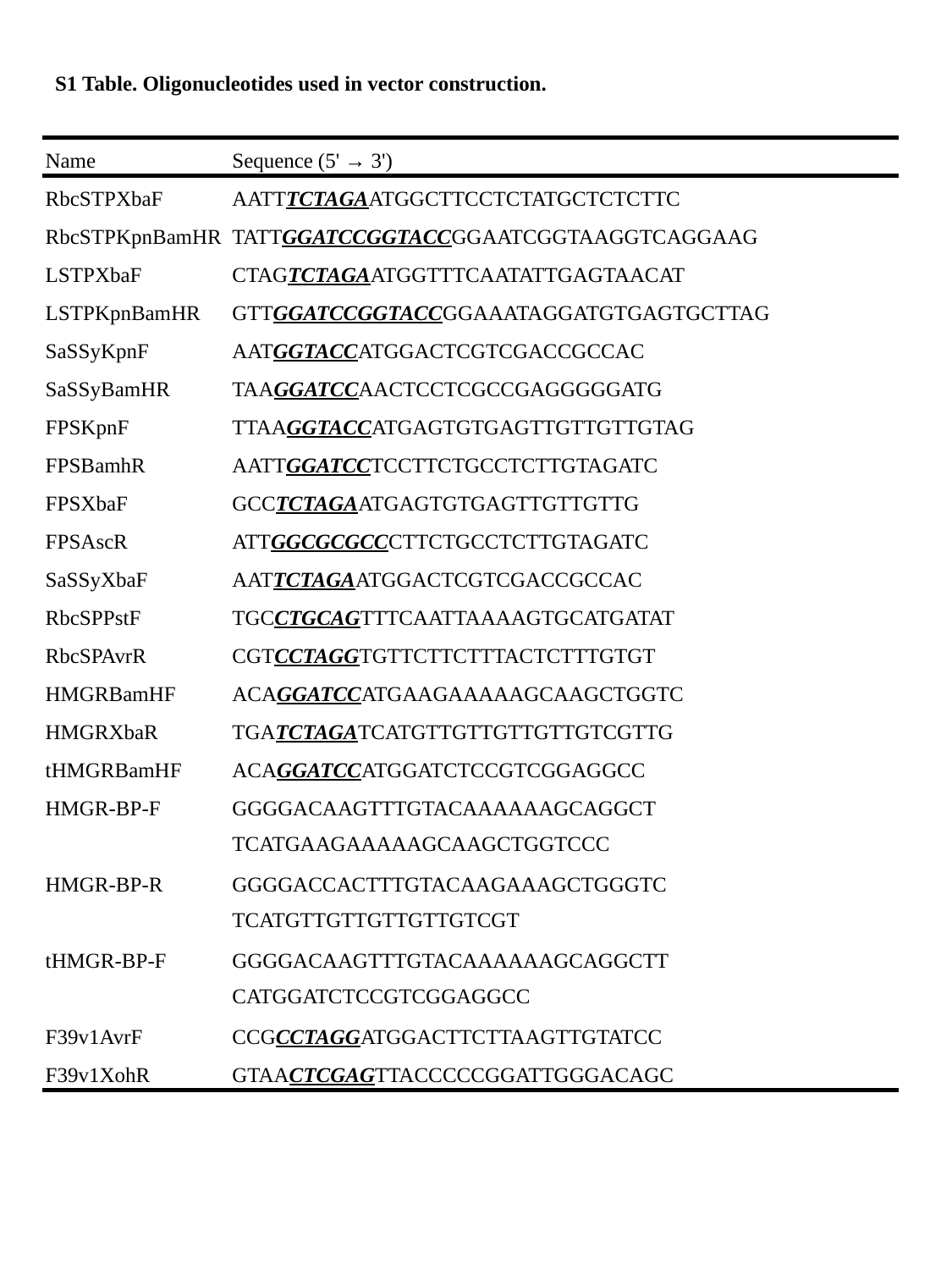

S1 Table. Oligonucleotides used in vector construction.
| Name | Sequence (5' → 3') |
| --- | --- |
| RbcSTPXbaF | AATTTCTAGAATGGCTTCCTCTATGCTCTCTTC |
| RbcSTPKpnBamHR | TATTGGATCCGGTACCGGAATCGGTAAGGTCAGGAAG |
| LSTPXbaF | CTAGTCTAGAATGGTTTCAATATTGAGTAACAT |
| LSTPKpnBamHR | GTTGGATCCGGTACCGGAAATAGGATGTGAGTGCTTAG |
| SaSSyKpnF | AATGGTACCATGGACTCGTCGACCGCCAC |
| SaSSyBamHR | TAAGGATCCAACTCCTCGCCGAGGGGGATG |
| FPSKpnF | TTAAGGTACCATGAGTGTGAGTTGTTGTTGTAG |
| FPSBamhR | AATTGGATCCTCCTTCTGCCTCTTGTAGATC |
| FPSXbaF | GCCTCTAGAATGAGTGTGAGTTGTTGTTG |
| FPSAscR | ATTGGCGCGCCCTTCTGCCTCTTGTAGATC |
| SaSSyXbaF | AATTCTAGAATGGACTCGTCGACCGCCAC |
| RbcSPPstF | TGCCTGCAGTTTCAATTAAAAGTGCATGATAT |
| RbcSPAvrR | CGTCCTAGGTGTTCTTCTTTACTCTTTGTGT |
| HMGRBamHF | ACAGGATCCATGAAGAAAAAGCAAGCTGGTC |
| HMGRXbaR | TGATCTAGATCATGTTGTTGTTGTTGTCGTTG |
| tHMGRBamHF | ACAGGATCCATGGATCTCCGTCGGAGGCC |
| HMGR-BP-F | GGGGACAAGTTTGTACAAAAAAGCAGGCT TCATGAAGAAAAAGCAAGCTGGTCCC |
| HMGR-BP-R | GGGGACCACTTTGTACAAGAAAGCTGGGTC TCATGTTGTTGTTGTTGTCGT |
| tHMGR-BP-F | GGGGACAAGTTTGTACAAAAAAGCAGGCTT CATGGATCTCCGTCGGAGGCC |
| F39v1AvrF | CCGCCTAGGATGGACTTCTTAAGTTGTATCC |
| F39v1XohR | GTAACTCGAGTTACCCCCGGATTGGGACAGC |
