# Supplementary material for: Production of santalenes and bergamotene in Nicotiana tabacum plants
Source: PLoS One. 2019 Jan 4;14(1):e0203249. doi: 10.1371/journal.pone.0203249 (PMC6319812; doi:10.1371/journal.pone.0203249)
Supplement: S2 Table — (PPTX) [file pone.0203249.s002.pptx]

## Slide 1
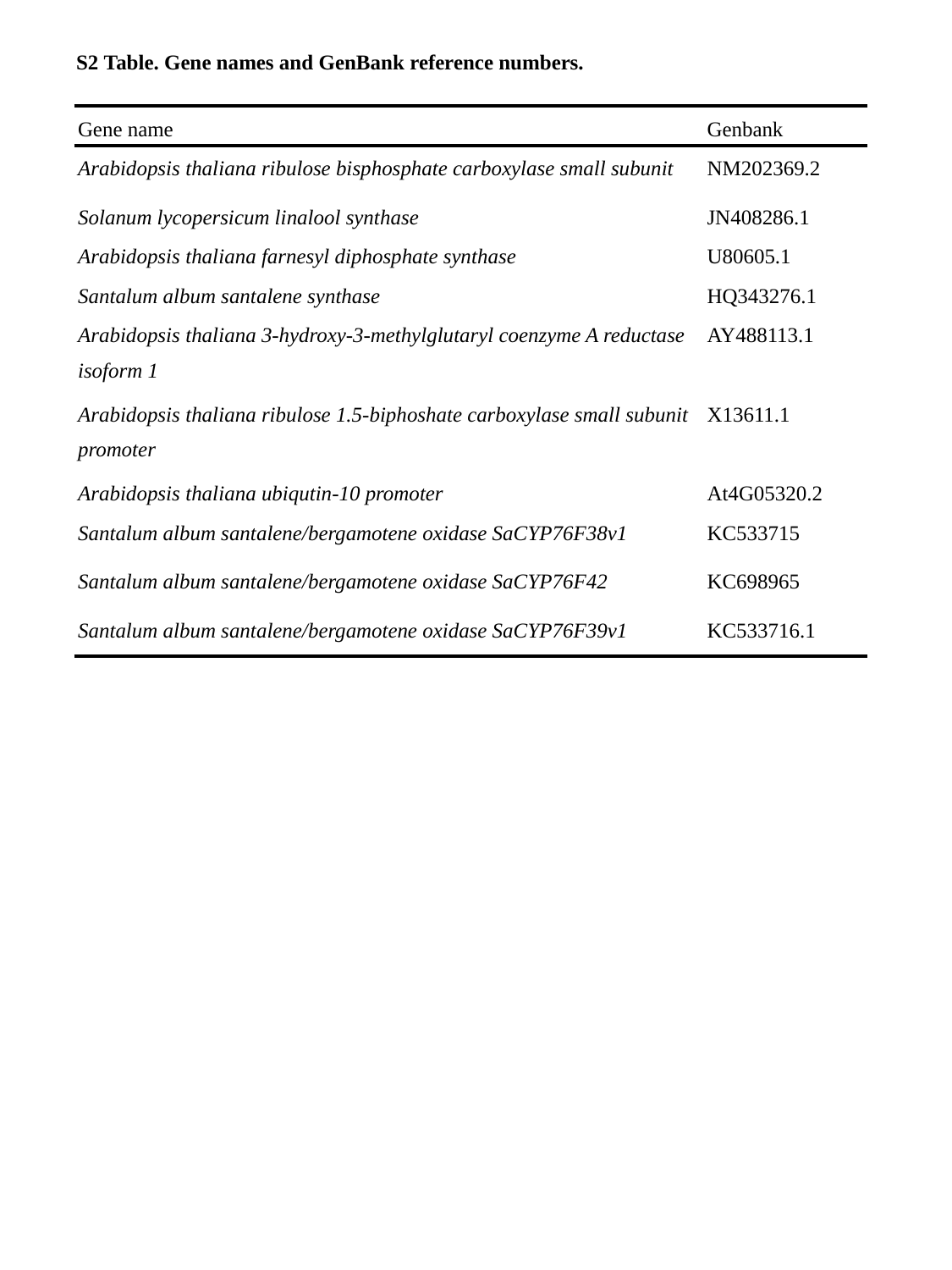

S2 Table. Gene names and GenBank reference numbers.
| Gene name | Genbank |
| --- | --- |
| Arabidopsis thaliana ribulose bisphosphate carboxylase small subunit | NM202369.2 |
| Solanum lycopersicum linalool synthase | JN408286.1 |
| Arabidopsis thaliana farnesyl diphosphate synthase | U80605.1 |
| Santalum album santalene synthase | HQ343276.1 |
| Arabidopsis thaliana 3-hydroxy-3-methylglutaryl coenzyme A reductase isoform 1 | AY488113.1 |
| Arabidopsis thaliana ribulose 1.5-biphoshate carboxylase small subunit promoter | X13611.1 |
| Arabidopsis thaliana ubiqutin-10 promoter | At4G05320.2 |
| Santalum album santalene/bergamotene oxidase SaCYP76F38v1 | KC533715 |
| Santalum album santalene/bergamotene oxidase SaCYP76F42 | KC698965 |
| Santalum album santalene/bergamotene oxidase SaCYP76F39v1 | KC533716.1 |
