# Supplementary material for: Production of santalenes and bergamotene in Nicotiana tabacum plants
Source: PLoS One. 2019 Jan 4;14(1):e0203249. doi: 10.1371/journal.pone.0203249 (PMC6319812; doi:10.1371/journal.pone.0203249)
Supplement: S3 Table — (PPTX) [file pone.0203249.s003.pptx]

## Slide 1
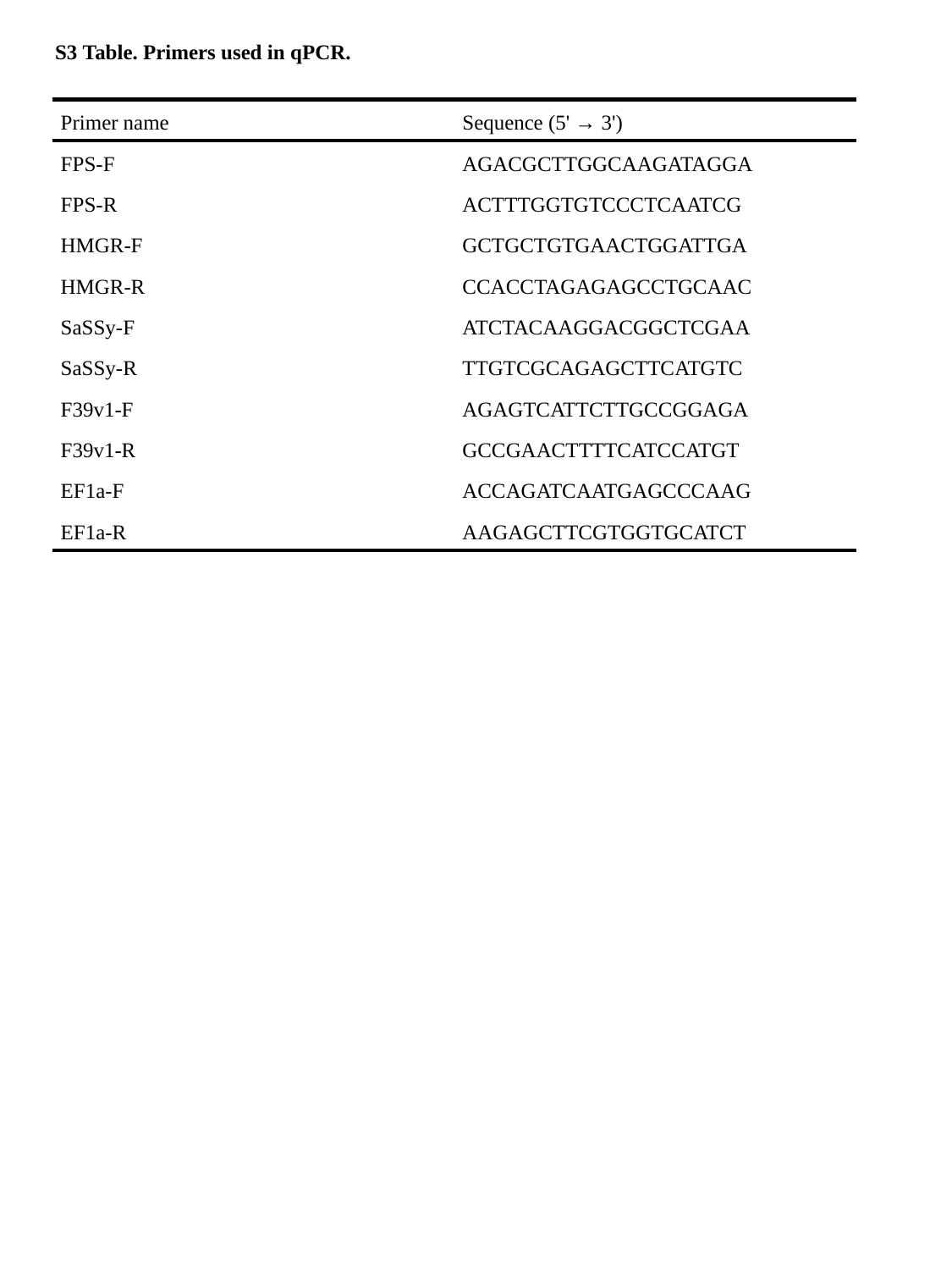

S3 Table. Primers used in qPCR.
| Primer name | Sequence (5' → 3') |
| --- | --- |
| FPS-F | AGACGCTTGGCAAGATAGGA |
| FPS-R | ACTTTGGTGTCCCTCAATCG |
| HMGR-F | GCTGCTGTGAACTGGATTGA |
| HMGR-R | CCACCTAGAGAGCCTGCAAC |
| SaSSy-F | ATCTACAAGGACGGCTCGAA |
| SaSSy-R | TTGTCGCAGAGCTTCATGTC |
| F39v1-F | AGAGTCATTCTTGCCGGAGA |
| F39v1-R | GCCGAACTTTTCATCCATGT |
| EF1a-F | ACCAGATCAATGAGCCCAAG |
| EF1a-R | AAGAGCTTCGTGGTGCATCT |
