# Supplementary material for: Production of santalenes and bergamotene in Nicotiana tabacum plants
Source: PLoS One. 2019 Jan 4;14(1):e0203249. doi: 10.1371/journal.pone.0203249 (PMC6319812; doi:10.1371/journal.pone.0203249)
Supplement: S1 Fig — (PPTX) [file pone.0203249.s004.pptx]

## Slide 1
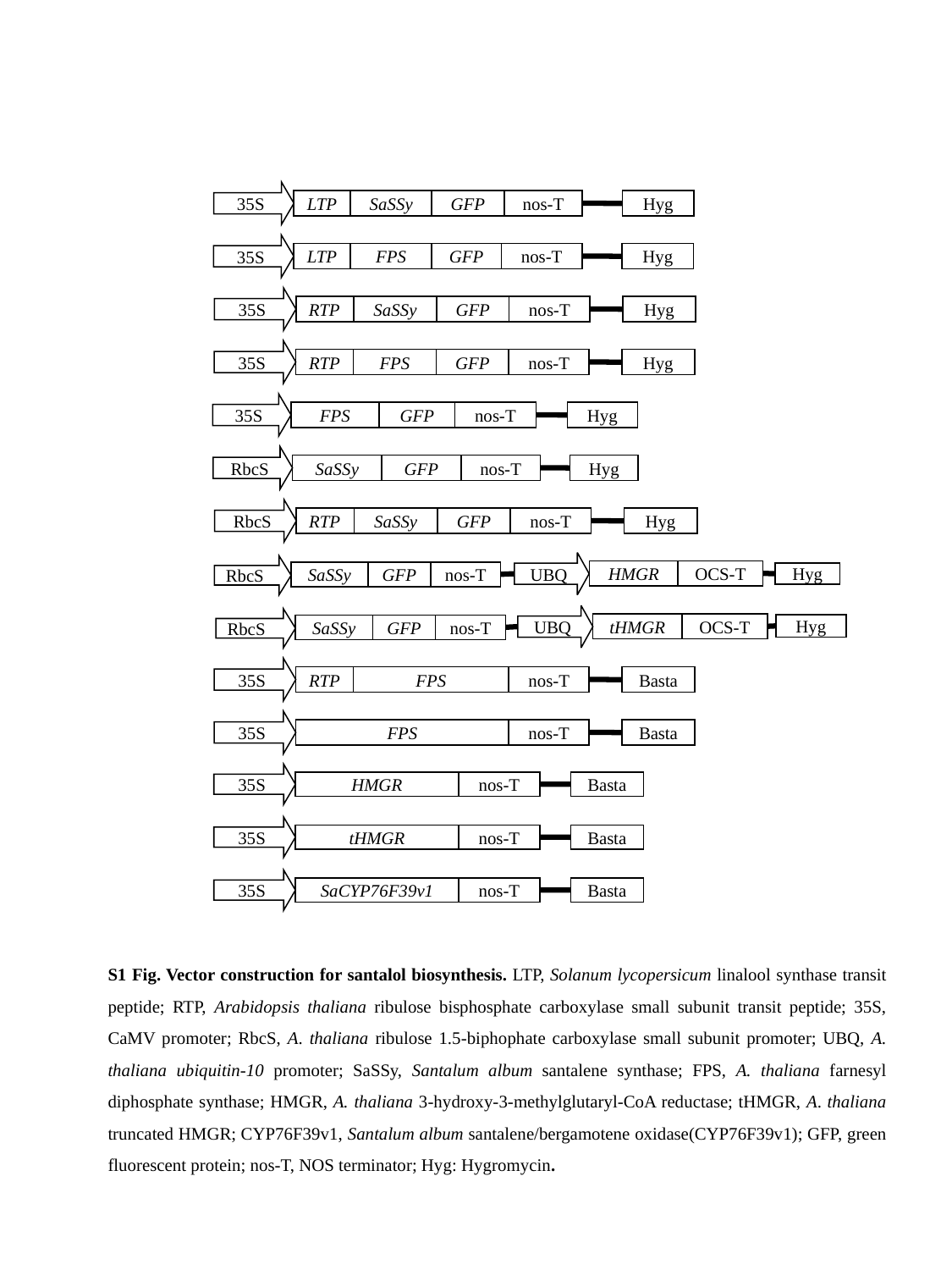

35S
LTP
SaSSy
GFP
nos-T
Hyg
35S
LTP
FPS
GFP
nos-T
Hyg
35S
RTP
SaSSy
GFP
nos-T
Hyg
35S
RTP
FPS
GFP
nos-T
Hyg
35S
FPS
GFP
nos-T
Hyg
RbcS
SaSSy
GFP
nos-T
Hyg
RbcS
RTP
SaSSy
GFP
nos-T
Hyg
UBQ
RbcS
HMGR
OCS-T
SaSSy
GFP
nos-T
Hyg
UBQ
RbcS
tHMGR
OCS-T
Hyg
SaSSy
GFP
nos-T
35S
RTP
FPS
nos-T
Basta
35S
FPS
nos-T
Basta
35S
HMGR
nos-T
Basta
35S
tHMGR
nos-T
Basta
35S
SaCYP76F39v1
nos-T
Basta
S1 Fig. Vector construction for santalol biosynthesis. LTP, Solanum lycopersicum linalool synthase transit peptide; RTP, Arabidopsis thaliana ribulose bisphosphate carboxylase small subunit transit peptide; 35S, CaMV promoter; RbcS, A. thaliana ribulose 1.5-biphophate carboxylase small subunit promoter; UBQ, A. thaliana ubiquitin-10 promoter; SaSSy, Santalum album santalene synthase; FPS, A. thaliana farnesyl diphosphate synthase; HMGR, A. thaliana 3-hydroxy-3-methylglutaryl-CoA reductase; tHMGR, A. thaliana truncated HMGR; CYP76F39v1, Santalum album santalene/bergamotene oxidase(CYP76F39v1); GFP, green fluorescent protein; nos-T, NOS terminator; Hyg: Hygromycin.
