# Supplementary material for: Production of santalenes and bergamotene in Nicotiana tabacum plants
Source: PLoS One. 2019 Jan 4;14(1):e0203249. doi: 10.1371/journal.pone.0203249 (PMC6319812; doi:10.1371/journal.pone.0203249)
Supplement: S2 Fig — (PPTX) [file pone.0203249.s005.pptx]

## Slide 1
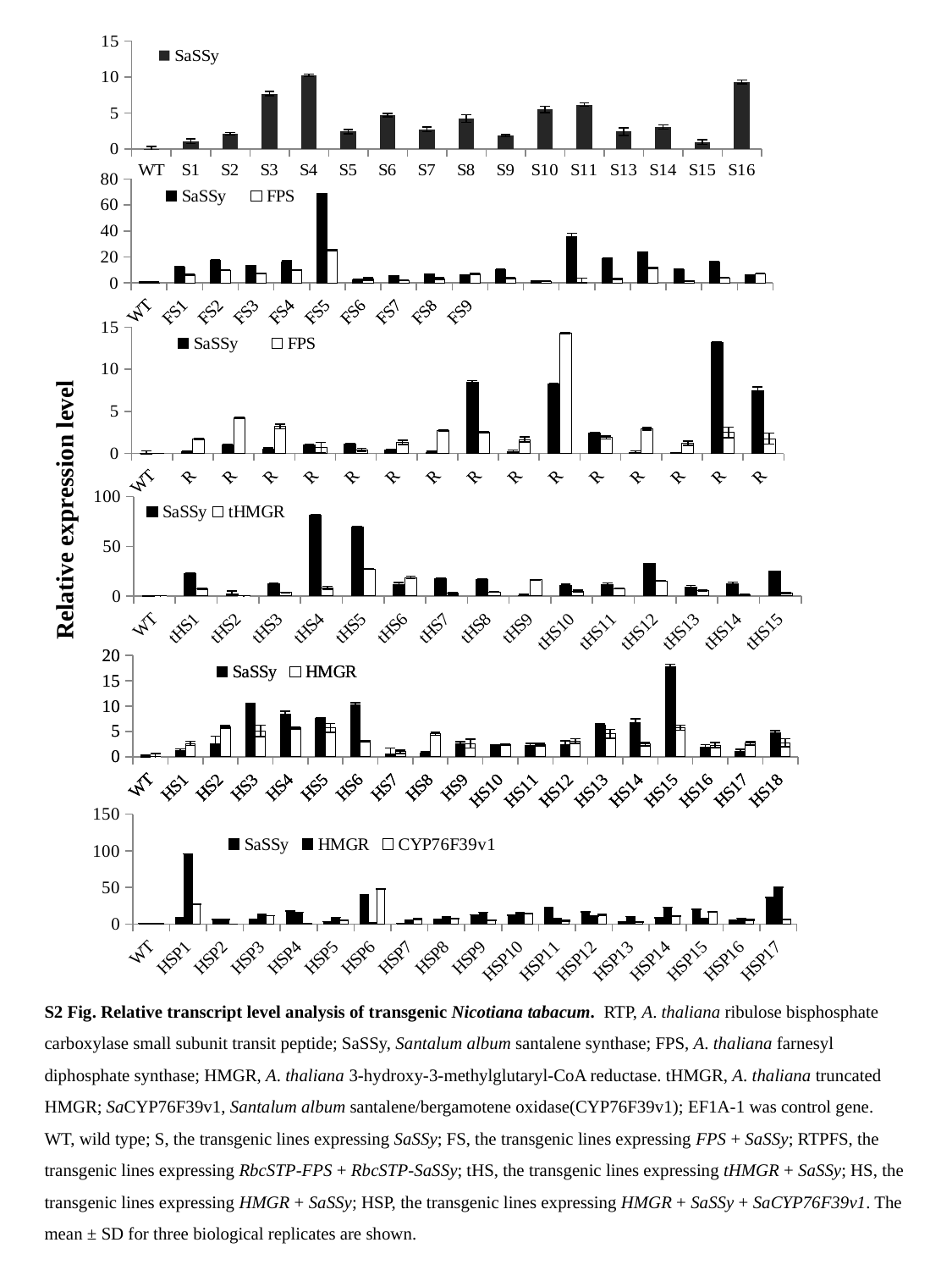

### Chart
| Category | SaSSy |
|---|---|
| WT | 0.020326157673093242 |
| S1 | 1.0998834584721138 |
| S2 | 2.141241718702654 |
| S3 | 7.70718006174484 |
| S4 | 10.269328902893998 |
| S5 | 2.4329494297324965 |
| S6 | 4.695416058373581 |
| S7 | 2.747061101668227 |
| S8 | 4.24255197256408 |
| S9 | 1.8722295524148966 |
| S10 | 5.491280725418198 |
| S11 | 6.169370687189534 |
| S13 | 2.4308080926059707 |
| S14 | 3.0746822693341667 |
| S15 | 1.0 |
| S16 | 9.327400078583125 |
### Chart
| Category | SaSSy | FPS |
|---|---|---|
| WT | 0.08332063627111334 | 0.010242180266531344 |
| FS1 | 12.242426815941824 | 6.234757005626332 |
| FS2 | 17.3174067478212 | 9.711315025717644 |
| FS3 | 13.527446839377877 | 7.457368674404783 |
| FS4 | 15.927901875833019 | 10.088688019458274 |
| FS5 | 68.75936581068245 | 25.410156104308445 |
| FS6 | 2.5411549239012907 | 2.947857256243795 |
| FS7 | 5.574404395617878 | 2.012050710860311 |
| FS8 | 6.6536731587489895 | 3.2746332398965112 |
| FS9 | 5.825913710183435 | 6.975677453837053 |
| FS10 | 10.479400878766572 | 3.5767930969683515 |
| FS11 | 1.5908718943114404 | 1.163657641335176 |
| FS12 | 35.624989076015126 | 0.5308342390591005 |
| FS13 | 18.889129779745012 | 2.9834880533829202 |
| FS14 | 23.568984046210993 | 11.626406356526822 |
| FS15 | 10.455216271435768 | 1.4773380635825522 |
| FS16 | 15.916865320975893 | 3.903225102421015 |
| FS17 | 5.92227301559597 | 7.059992577216665 |
### Chart
| Category | SaSSy | FPS |
|---|---|---|
| WT | 0.00013092951715225657 | 0.010233146794827522 |
| RTPFS1 | 0.20466046351731118 | 1.7125649055158392 |
| RTPFS2 | 1.0 | 4.235723257272571 |
| RTPFS3 | 0.47954241584482304 | 3.199150062819345 |
| RTPFS4 | 1.0419184281271876 | 0.7198678536365879 |
| RTPFS5 | 1.119225186223999 | 0.4366444778645339 |
| RTPFS6 | 0.39150536967874267 | 1.3196243766584388 |
| RTPFS7 | 0.1364450220597136 | 2.724179309265729 |
| RTPFS8 | 8.465815220786359 | 2.517756914802985 |
| RTPFS9 | 0.23125738350385244 | 1.6582467135965222 |
| RTPFS10 | 8.237803233331968 | 14.277716276290002 |
| RTPFS11 | 2.3797725072545197 | 1.8873896977149462 |
| RTPFS12 | 0.13898499102665934 | 2.9310786217666127 |
| RTPFS13 | 0.12408546177477418 | 1.2335238982784356 |
| RTPFS14 | 13.153057842872679 | 2.517369515514263 |
| RTPFS15 | 7.4928505003565755 | 1.7712311504790614 |
### Chart
| Category | SaSSy | tHMGR |
|---|---|---|
| WT | 0.1611881584668402 | 0.0344385131343931 |
| tHS1 | 22.86629941697039 | 7.108277481136743 |
| tHS2 | 1.8766388015436255 | 0.17565704365621684 |
| tHS3 | 12.744630723462619 | 3.493080764318267 |
| tHS4 | 81.78802190783148 | 8.153955028760617 |
| tHS5 | 69.34969664350366 | 27.20563002288099 |
| tHS6 | 11.649135587576597 | 18.663745912554187 |
| tHS7 | 17.389577789229673 | 2.7229427815148783 |
| tHS8 | 17.01990406514523 | 4.239321359691243 |
| tHS9 | 0.5488501947146416 | 16.425135115895515 |
| tHS10 | 10.64042840972079 | 5.0 |
| tHS11 | 11.30966533001407 | 7.671453770551342 |
| tHS12 | 32.638081086963425 | 15.093268270880568 |
| tHS13 | 9.299486142012253 | 5.4220308934464745 |
| tHS14 | 12.508333339007956 | 1.3712579644320406 |
| tHS15 | 24.803715354661886 | 2.7756647621412402 |Relative expression level
### Chart
| Category | SaSSy | HMGR |
|---|---|---|
| WT | 0.011315674032348531 | 0.003610831966498479 |
| HS1 | 1.2970595043343822 | 2.6223174296495033 |
| HS2 | 2.4999518933555978 | 5.8990119565653645 |
| HS3 | 10.269328902893998 | 5.08243549548752 |
| HS4 | 8.484694215685039 | 5.647719092595699 |
| HS5 | 7.607385246903412 | 5.663114371778497 |
| HS6 | 10.176477267804277 | 3.049404778556861 |
| HS7 | 0.5543538257479727 | 1.0 |
| HS8 | 0.7601190693772365 | 4.4888752407296995 |
| HS9 | 2.56865357682979 | 2.5645292034011242 |
| HS10 | 2.021742603410356 | 2.4503174457384014 |
| HS11 | 2.2394860300498927 | 2.4116541025935967 |
| HS12 | 2.39920483270248 | 3.0981145769168412 |
| HS13 | 6.1975284969274345 | 4.5193438689882885 |
| HS14 | 6.707180061744835 | 2.4809712206221812 |
| HS15 | 17.80073614986089 | 5.730188770398647 |
| HS16 | 1.9460957131157381 | 2.274485125758246 |
| HS17 | 1.0957016359509097 | 2.641883166121899 |
| HS18 | 4.79161612661667 | 2.7525046480194892 |
### Chart
| Category | SaSSy | HMGR |
|---|---|---|
| WT | 0.011315674032348531 | 0.0036108319664984816 |
| HS1 | 1.2970595043343822 | 2.6223174296495033 |
| HS2 | 2.4999518933555978 | 5.8990119565653645 |
| HS3 | 10.269328902893998 | 5.08243549548752 |
| HS4 | 8.484694215685051 | 5.647719092595699 |
| HS5 | 7.607385246903406 | 5.663114371778489 |
| HS6 | 10.176477267804282 | 3.049404778556861 |
| HS7 | 0.5543538257479722 | 1.0 |
| HS8 | 0.7601190693772365 | 4.4888752407296995 |
| HS9 | 2.56865357682979 | 2.5645292034011242 |
| HS10 | 2.021742603410356 | 2.4503174457384014 |
| HS11 | 2.2394860300498927 | 2.4116541025935967 |
| HS12 | 2.39920483270248 | 3.0981145769168412 |
| HS13 | 6.1975284969274345 | 4.5193438689882885 |
| HS14 | 6.707180061744835 | 2.4809712206221812 |
| HS15 | 17.80073614986089 | 5.730188770398647 |
| HS16 | 1.9460957131157381 | 2.2744851257582432 |
| HS17 | 1.095701635950909 | 2.641883166121899 |
| HS18 | 4.79161612661667 | 2.7525046480194892 |
### Chart
| Category | SaSSy | HMGR | CYP76F39v1 |
|---|---|---|---|
| WT | 0.09507112219716427 | 0.05102447818032946 | 0.19264277708960217 |
| HSP1 | 9.15452057313597 | 95.82520892164179 | 26.834181902109837 |
| HSP2 | 6.024400431584037 | 6.797479993109183 | 0.01838491945991542 |
| HSP3 | 5.9045113407808385 | 14.054887491813739 | 11.538096530092227 |
| HSP4 | 17.39761533736493 | 16.30225914586749 | 0.07020252183880651 |
| HSP5 | 3.308439734238777 | 9.496088190055506 | 5.5174436873082495 |
| HSP6 | 39.391642445549394 | 2.1802597139395314 | 47.69722186012941 |
| HSP7 | 1.1800251978006244 | 5.998769786891249 | 7.087777587998189 |
| HSP8 | 6.854973077896878 | 10.717030667973768 | 7.8010410607754626 |
| HSP9 | 12.854049249655572 | 16.40806643661944 | 5.355421027913765 |
| HSP10 | 11.751863726749963 | 16.61022832868904 | 14.719601067081738 |
| HSP11 | 22.956291066236993 | 7.844419376785538 | 4.518988858280443 |
| HSP12 | 16.95906036973333 | 11.360858868347425 | 12.611488575667774 |
| HSP13 | 2.76219977682328 | 10.386708705292548 | 3.082996674633364 |
| HSP14 | 8.61082220474421 | 23.311985822368243 | 10.543875484980948 |
| HSP15 | 19.956926758276268 | 8.339726086728977 | 17.07720540863939 |
| HSP16 | 4.914862310028744 | 8.303195497183703 | 5.731846601492403 |
| HSP17 | 36.373545824797674 | 50.02809688001816 | 6.3247129328315355 |S2 Fig. Relative transcript level analysis of transgenic Nicotiana tabacum. RTP, A. thaliana ribulose bisphosphate carboxylase small subunit transit peptide; SaSSy, Santalum album santalene synthase; FPS, A. thaliana farnesyl diphosphate synthase; HMGR, A. thaliana 3-hydroxy-3-methylglutaryl-CoA reductase. tHMGR, A. thaliana truncated HMGR; SaCYP76F39v1, Santalum album santalene/bergamotene oxidase(CYP76F39v1); EF1A-1 was control gene. WT, wild type; S, the transgenic lines expressing SaSSy; FS, the transgenic lines expressing FPS + SaSSy; RTPFS, the transgenic lines expressing RbcSTP-FPS + RbcSTP-SaSSy; tHS, the transgenic lines expressing tHMGR + SaSSy; HS, the transgenic lines expressing HMGR + SaSSy; HSP, the transgenic lines expressing HMGR + SaSSy + SaCYP76F39v1. The mean ± SD for three biological replicates are shown.
