# Supplementary material for: Production of santalenes and bergamotene in Nicotiana tabacum plants
Source: PLoS One. 2019 Jan 4;14(1):e0203249. doi: 10.1371/journal.pone.0203249 (PMC6319812; doi:10.1371/journal.pone.0203249)
Supplement: S3 Fig — (PPTX) [file pone.0203249.s006.pptx]

## Slide 1
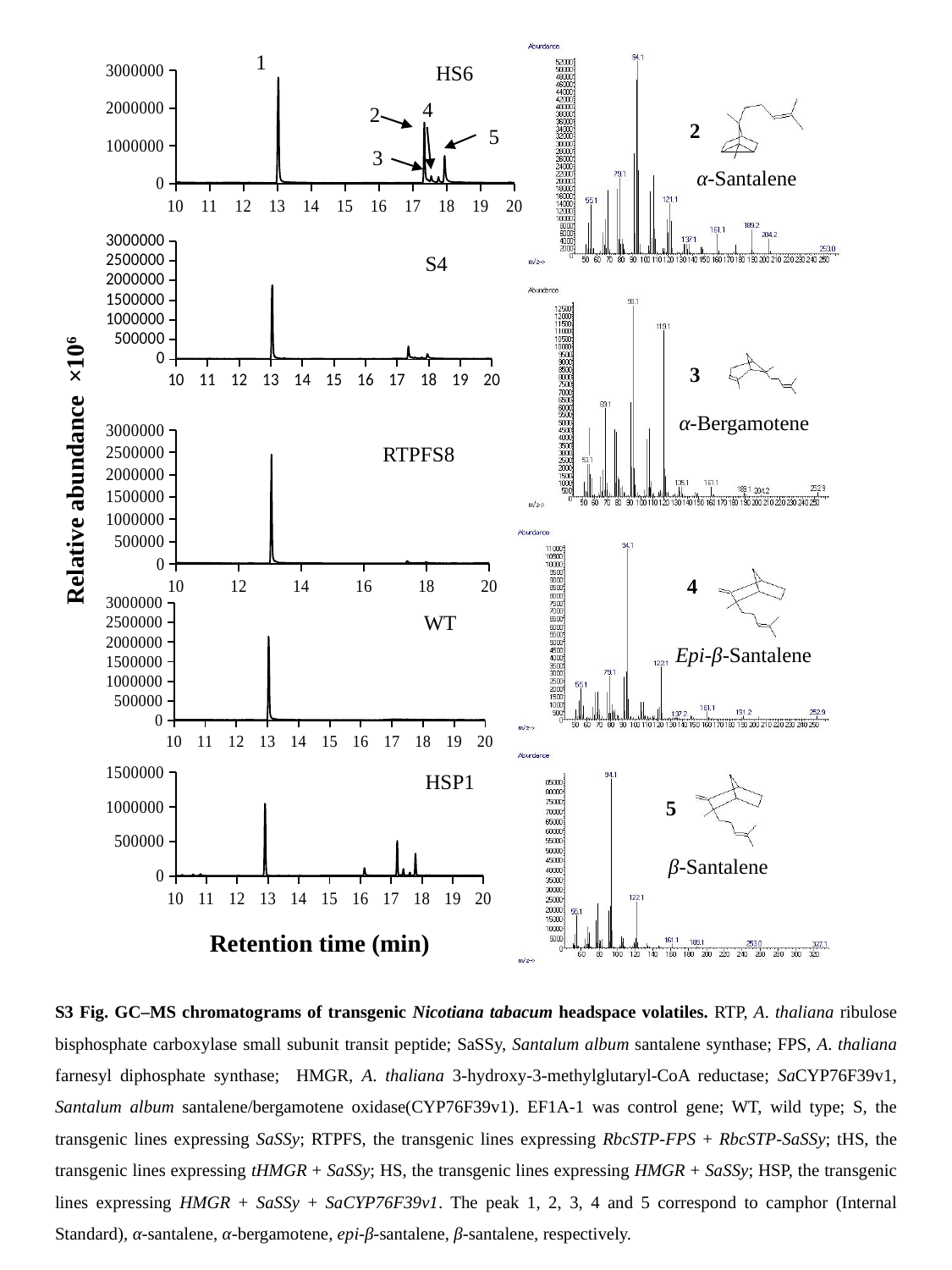

1
### Chart
| Category | |
|---|---|HS6
4
2
2
5
3
α-Santalene
### Chart
| Category | |
|---|---|S4
3
α-Bergamotene
### Chart
| Category | |
|---|---|RTPFS8
Relative abundance ×106
4
### Chart
| Category | |
|---|---|WT
Epi-β-Santalene
### Chart
| Category | |
|---|---|
HSP1
5
β-Santalene
 Retention time (min)
S3 Fig. GC–MS chromatograms of transgenic Nicotiana tabacum headspace volatiles. RTP, A. thaliana ribulose bisphosphate carboxylase small subunit transit peptide; SaSSy, Santalum album santalene synthase; FPS, A. thaliana farnesyl diphosphate synthase; HMGR, A. thaliana 3-hydroxy-3-methylglutaryl-CoA reductase; SaCYP76F39v1, Santalum album santalene/bergamotene oxidase(CYP76F39v1). EF1A-1 was control gene; WT, wild type; S, the transgenic lines expressing SaSSy; RTPFS, the transgenic lines expressing RbcSTP-FPS + RbcSTP-SaSSy; tHS, the transgenic lines expressing tHMGR + SaSSy; HS, the transgenic lines expressing HMGR + SaSSy; HSP, the transgenic lines expressing HMGR + SaSSy + SaCYP76F39v1. The peak 1, 2, 3, 4 and 5 correspond to camphor (Internal Standard), α-santalene, α-bergamotene, epi-β-santalene, β-santalene, respectively.
