# Supplementary material for: Production of santalenes and bergamotene in Nicotiana tabacum plants
Source: PLoS One. 2019 Jan 4;14(1):e0203249. doi: 10.1371/journal.pone.0203249 (PMC6319812; doi:10.1371/journal.pone.0203249)
Supplement: S4 Fig — (PPTX) [file pone.0203249.s007.pptx]

## Slide 1
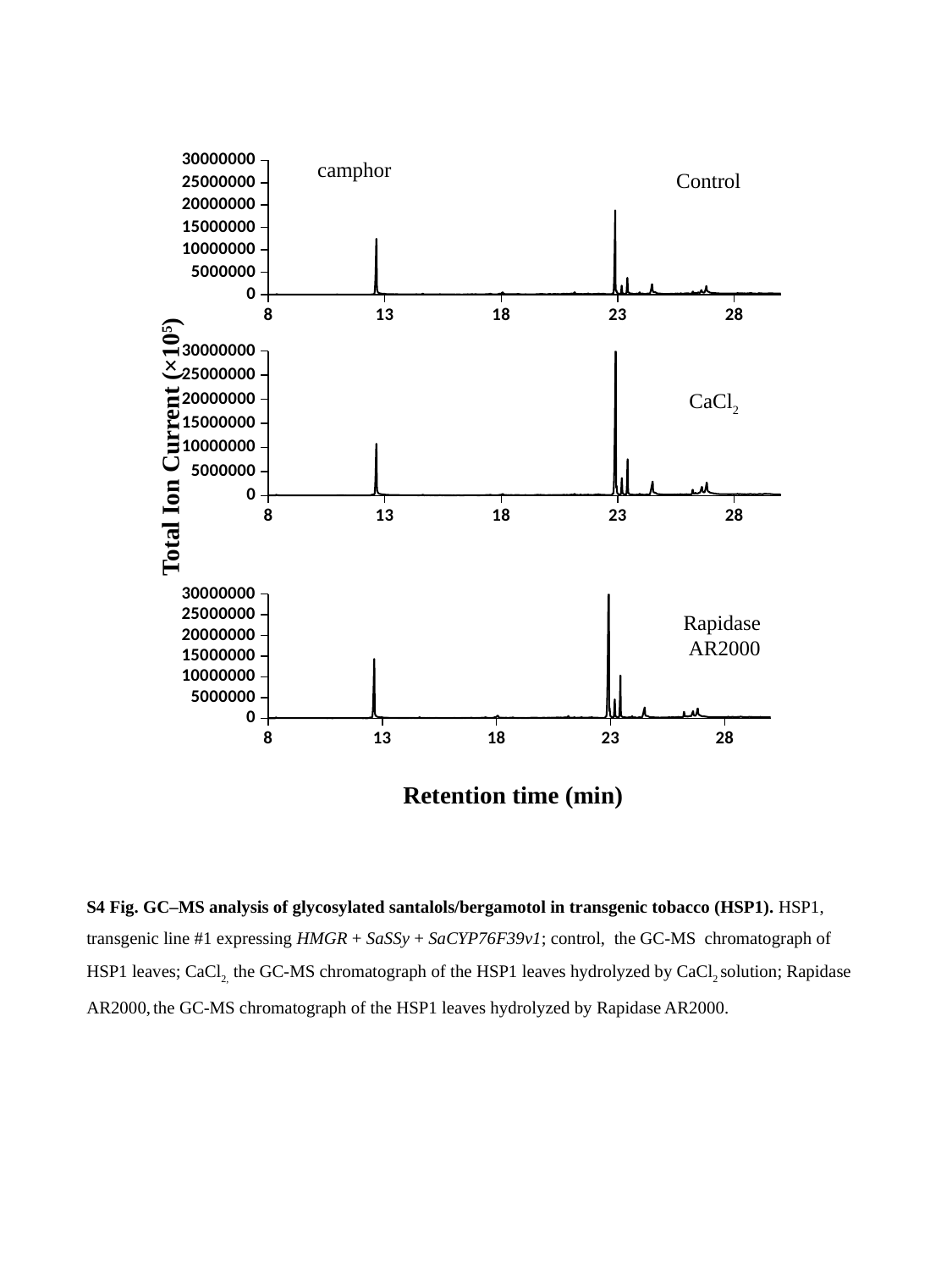

### Chart
| Category | |
|---|---|camphor
Control
### Chart
| Category | |
|---|---|CaCl2
 Total Ion Current (×105)
### Chart
| Category | |
|---|---|Rapidase
AR2000
Retention time (min)
S4 Fig. GC–MS analysis of glycosylated santalols/bergamotol in transgenic tobacco (HSP1). HSP1, transgenic line #1 expressing HMGR + SaSSy + SaCYP76F39v1; control, the GC-MS chromatograph of HSP1 leaves; CaCl2, the GC-MS chromatograph of the HSP1 leaves hydrolyzed by CaCl2 solution; Rapidase AR2000, the GC-MS chromatograph of the HSP1 leaves hydrolyzed by Rapidase AR2000.
