# Supplementary material for: Production of santalenes and bergamotene in Nicotiana tabacum plants
Source: PLoS One. 2019 Jan 4;14(1):e0203249. doi: 10.1371/journal.pone.0203249 (PMC6319812; doi:10.1371/journal.pone.0203249)
Supplement: S5 Fig — (PPTX) [file pone.0203249.s008.pptx]

## Slide 1
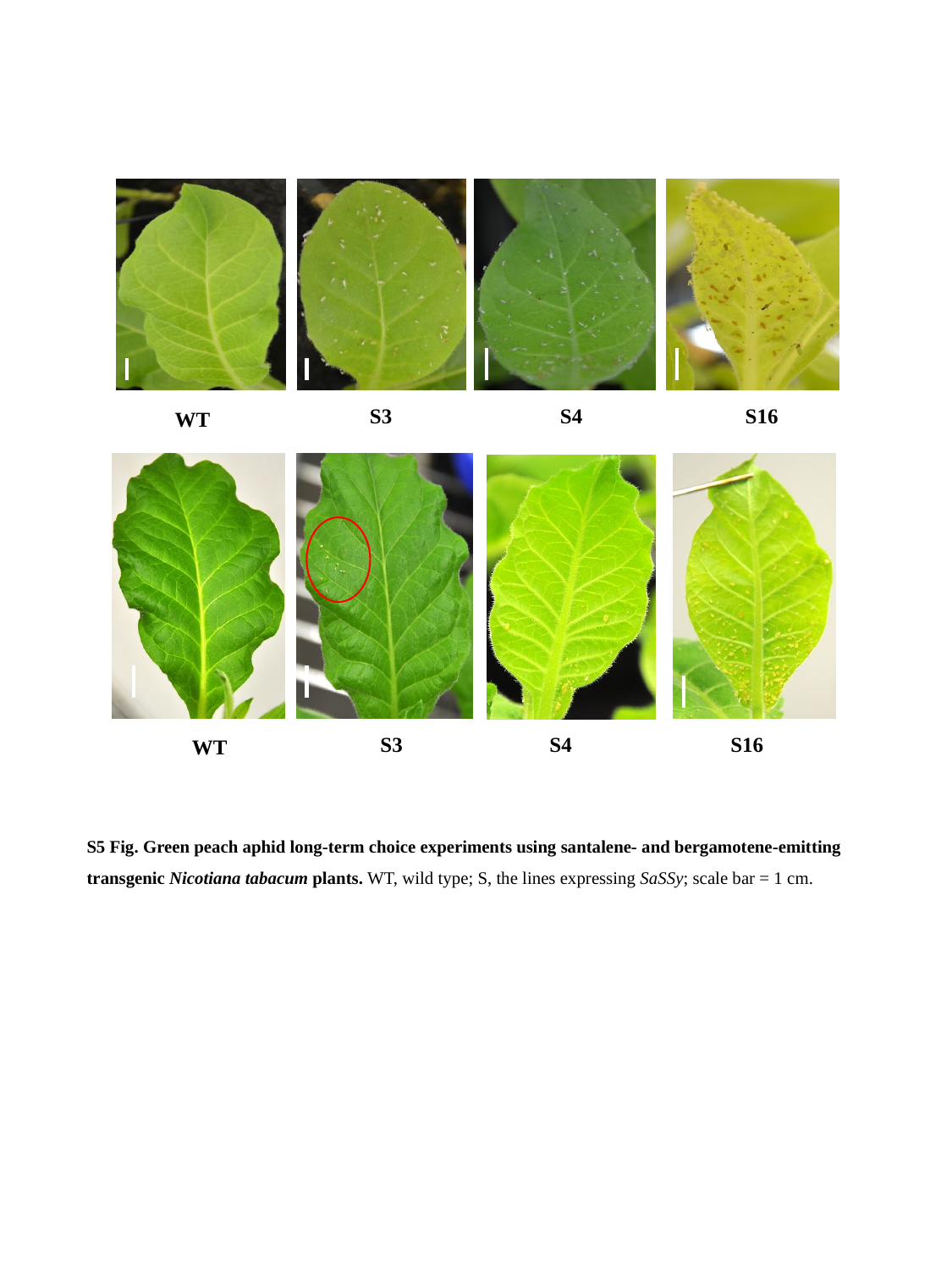

WT
S3
S4
S16
WT
S3
S4
S16
S5 Fig. Green peach aphid long-term choice experiments using santalene- and bergamotene-emitting transgenic Nicotiana tabacum plants. WT, wild type; S, the lines expressing SaSSy; scale bar = 1 cm.
