# Supplementary material for: Production of santalenes and bergamotene in Nicotiana tabacum plants
Source: PLoS One. 2019 Jan 4;14(1):e0203249. doi: 10.1371/journal.pone.0203249 (PMC6319812; doi:10.1371/journal.pone.0203249)
Supplement: S6 Fig — (PPTX) [file pone.0203249.s009.pptx]

## Slide 1
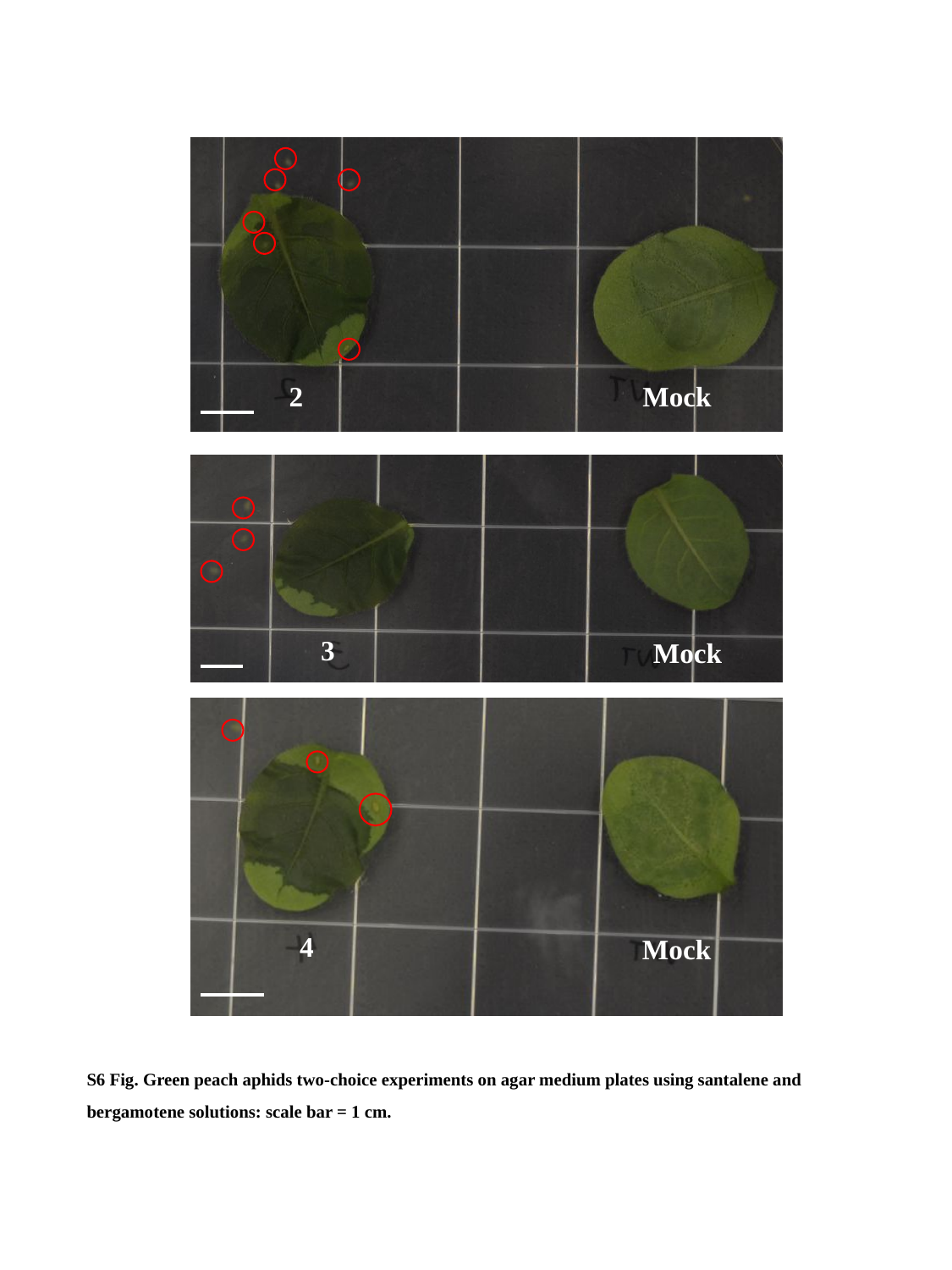

2
Mock
3
Mock
4
Mock
S6 Fig. Green peach aphids two-choice experiments on agar medium plates using santalene and bergamotene solutions: scale bar = 1 cm.
